# A simple quantitative method using TLC-image analysis to determine fructooligosaccharides (FOS) in food samples

**DOI:** 10.55730/1300-0527.3436

**Published:** 2022-05-06

**Authors:** Diana Beatriz MUÑİZ-MÁRQUEZ, Marco Antonio MARTÍNEZ-CERVANTES, Alaín MARTÍNEZ-PÉREZ, Cristóbal Noé AGUİLAR, Pedro AGUİLAR-ZARATE, Jorge Enrique WONG-PAZ

**Affiliations:** 1 Engineering Department, Technological Institute of Ciudad Valles, National Technological Institute of Mexico, Ciudad Valles, S.L.P., Mexico; 2 Bioprocesses & Bioproducts Research Group. BBG-DIA. Food Research Department, School of Chemistry, Universidad Autónoma de Coahuila, Saltillo, Coahuila, Mexico; 3 Faculty of Professional Studies Huasteca Zone, Autonomous University of San Luis Potosí, Ciudad Valles S.L.P., Mexico

**Keywords:** Quantitative-TLC, image analysis, fructooligosaccharides determination, functional foods

## Abstract

The thin-layer chromatography technique (TLC) is a simple and inexpensive analysis commonly used to identify qualitatively the presence of carbohydrates in food samples such as mono- di and oligosaccharides particularly. TLC assay could be improved using image processing software for the semiquantitative determination of this type of compound. In the present work, TLC-image analysis with Silica Gel 60 TLC plates was used for the semiquantitative determination of 6 standards of carbohydrates (glucose, fructose, sucrose, 1-kestose, nystose, and fructofuranosylnystose). Subsequently, the areas of the spots of each compound were determined by digitizing in a conventional office scanner. Then, the segmentation of the images is carried out using software for image processing. The calibration curves were plotted in the Excel software using the average of the areas of the pigmentations obtained in pixels. In this study, the technique of thin-layer chromatography was also used to quantitatively determine the presence of carbohydrates in food samples such as honey, garlic, and onion. Values of determination coefficient (R2) greater than 0.97 in all the calibration curves were obtained. This technique could be useful for detecting carbohydrates (monosaccharides, disaccharides, and oligosaccharides) in analytical assays and food samples without needing specialized analytical equipment. In this work, it was possible to determine the concentration of carbohydrates in samples of garlic and onion that showed the presence of prebiotic carbohydrates in addition to sucrose, glucose, and fructose.

## 1. Introduction

Functional foods are conventional products consumed as a part of daily life that provide specifical benefits to health over and above the normal nutritional value they provide, such as nutrients and energy [1, 2]. These foods were introduced in Japan in the 1980s and are products fortified with specific ingredients with physiological health effects such as antioxidants, vitamins, and prebiotics [3].

According to the International Scientific Association for Probiotics and Prebiotics (ISAPP), prebiotics are “substrates that are selectively utilized by host microorganisms conferring a health benefit” [4]. Prebiotics are nondigestible substrates considered beneficial compounds for the body because they present different biological properties being one of the most important contributions to the growth and proliferation of a limited number of probiotic bacteria that compete with the pathogenic microbiota in the intestinal area [2, 3]. Also, they not only help to maintain the health of intestinal microbiota, but also act as therapeutic agents against other diseases such as enhancing mineral absorption such as calcium, iron, and zinc, decreasing the risk of colon cancer, the levels of triglycerides and cholesterol in the human body [6]. Prebiotic carbohydrates are present in natural sources such as Jerusalem artichoke, garlic, onion, asparagus, chicory, tomatoes, banana, and honey but in a low concentration [7]. They could be recovered, purified, and concentrated to be used as functional ingredients for products developed in the food industry [5, 6]. Several new products indicating the presence of functional prebiotics are appearing in the market, but sometimes they are not quantified, and only they are reported because contain a raw material previously reported with the presence of these compounds. However, changes that could occur in the quantity and chemical composition during food processing are not considered. Therefore, today the quantification of prebiotics in natural sources, foods, and beverages through a simple, economical, and fast method is a very important subject.

Nowadays, specialized analytical equipment for the quantification of prebiotics and other common carbohydrates is expensive and inaccessible. Liquid chromatography coupled to mass spectrometry (LC-MS) [10], gas chromatography (GC) [11], and MALDI-TOF-MS analysis [12] are commonly used in this sense.

Hence, it is convenient to find simple and low-cost alternatives to the analytical equipment for the quantification of prebiotics. An alternative to this problem is the determination and quantification of carbohydrates by thin-layer chromatography (TLC) because it is a reliable and quick technique. In this method, the sample to be analyzed is deposited near one end of an aluminum plate precoated with a thin layer of adsorbent (stationary phase as Silica Gel 60). So, the sheet is placed in a closed cuvette that contains a solvent system (eluent or mobile phase). As the solvent mixture rises by capillarity through the adsorbent, it produced a differential distribution of the products present in the sample between the solvent and the adsorbent. The two adsorbents most widely used in TLC as a stationary phase are silica gel (SiO_2_) and alumina (Al_2_O_3_), both of polar character. The adsorbent must be inert with the substances to be analyzed and not act as a catalyst in decomposition reactions. The TLC has been used only for the qualitative determination of prebiotics [13].

Although TLC has already been used quantitatively, compared to the qualitative use it has limited applications. This deficiency of use is mainly due to the difficulty of finding signals that may be related to the concentration of calibration standards and samples [14]. The main method for quantitative TLC is for scanning densitometry based on measuring the fluorescence or absorbance [15]. Another method used is image processing software tools to obtain an analytical response usually based on the RGB color system [14]. The method proposed in this work is the quantification by pixel area with the use of an image processing software. The pixel area can be used as an analytical signal for the quantification of carbohydrates (monosaccharides, disaccharides, and oligosaccharides) in TLC, because the spot size and color intensity increase with carbohydrate concentration. ImageJ software is effective for this purpose because it allows segmentation of an image defined by the user and to calculate the area (expressed in pixel) with greater ease and precision, it is also a public domain digital image processing program ( https://imagej.nih.gov/ij/ ). The purpose of this study was to standardize the TLC method coupled to the image analysis to convert it into a quantitative analysis by means of calculating the area of the spots expressed in pixel values and subsequent construction of calibration curves with the corresponding standards.

## 2. Materials and methods

### 2.1. Chemicals and prebiotic standards

The standards of sucrose, fructose, and glucose were purchased from Sigma Aldrich and the standards of prebiotics 1-kestose (99% purity), nystose (99% purity), and fructofuranosylnystose (99% purity) were obtained from Wako Pure Chemical Industries (Tokyo, Japan). Silica gel 60 F_254_ TCL plates were purchased from Merck company. All solvents were reactive grade. Standard curves of each one of the carbohydrates were prepared as follows: 0.10, 0.20, 0.25, 0.30, 0.35, 0.40, 0.45, and 0.50 g/L, and then keep them in freezing (–4 °C) until use.

### 2.2. Thin-layer chromatography (TLC)

For the marking of the silica-gel plate, the technique reported by Mellado-Mojica and López (2015) [16] was followed. In the line of application, the points were marked according to the samples that were to be run 1.5 cm of separation between each application point was used. In the application points, 5 μL of each concentration of the standard curves prepared for each carbohydrate standard were placed. In the mobile phase a system solvent with 96 mL of propanol, 32 mL of distilled water, and 24 mL of butanol were used [17].

The revealer for TLC was prepared by developing a solution of aniline (4% ) in 50 mL of acetone and a diphenylamine (4%) dilution in 50 mL of acetone. These dilutions were mixed with 10 mL of phosphoric acid until homogenized [13, 15]. The mixture was kept refrigerated (4–6 °C) in an amber bottle.

The elution chamber was filled with the mobile phase so that this mixture will not exceed the line with the application points of the prebiotic standards. The TLC plate was introduced with the standards previously placed and the wetting process was carried out by capillarity. The plate was introduced and waited for it to reach the first mark. Once the first mark was reached, the plate was removed and allowed to dry. When it was dry, it was reintroduced and watched until it reached the next mark, it was removed again, and it was waited until it dried and then introduced again. This operation was repeated until reaching the final mark.

**Figure 1:**
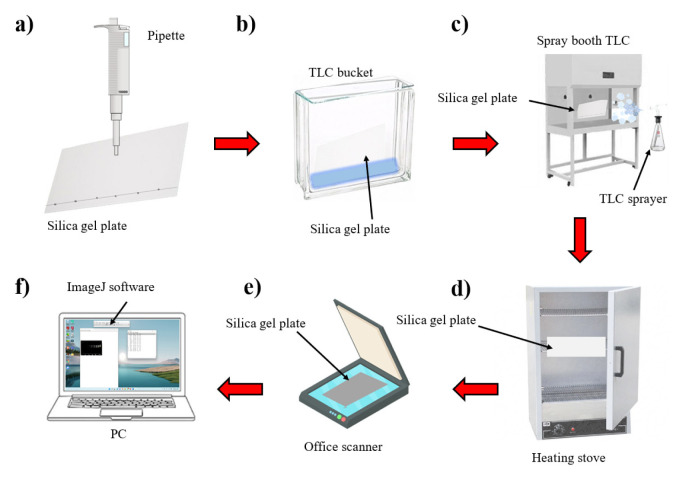
**A** general outline of the TLC methodology for semiquantitative carbohydrate analysis using an office scanner together with an image processing software. (a) Placement of samples using a pipette with 5 μL of each standard. (b) Wetting by capillarity with mobile phase. (c) Revealed the plates in a spray booth. (d) Heating of the plates for the revealed of the spots. (e) Scan the plates with a flat surface scanner. (f) Semiquantification of carbohydrates with the image processing software.

After having reached the last mark in capillary wetting, the plates were dried and placed in support inside an extraction hood and then were sprayed with a reagent previously prepared (diphenylamine and aniline in acetone 4% and phosphoric acid) with the help of a sprayer and a vacuum pump. The resulting plates were collocated in an oven at a temperature of 90 °C for 5 min, to reveal the corresponding spots of carbohydrate standards (Figure 1).

### 2.3. Image analysis of the TLC plates

The analysis began immediately after revealing the silica gel plates in the oven. For this, the revealed plates were scanned using a conventional office scanner (HP DeskJet 1510) with a resolution of 600 dpi and 2400 × 2400 dpi. Once the plates were scanned, the image processing was carried out in the ImageJ software, where the area of interest could be obtained for each spot. A format change was made to 16 bits, the sharpness of the image was adjusted, and the threshold limit was inserted to carry out the segmentation of the carbohydrate spots. Subsequently, the corresponding area of each one was estimated. With data of the areas obtained in pixels, the respective calibration curves were constructed and analyzed with Microsoft Excel 2013 software. The regression coefficient (R^2^) and the trendline were also obtained.

### 2.4. Quantitative analysis of functional prebiotics in food samples

In order to show the usefulness of the assay, some food samples reported to contain functional carbohydrates were analyzed and quantified under the developed method.

The samples analyzed were honey (diluted 1:100 in distilled water), garlic powder (diluted 1:15), and onion juice (obtained from one onion of 200 g from vegetal material). All the samples were filtrated to remove larger impurities. For the quantification of carbohydrates present, 5 μL of each sample were placed in the application points of the silica gel plate following the TLC technique described above. A segmentation of the plates was carried out with the ImageJ software. The areas in pixels of the spots of each sample were determined and their respective concentrations were calculated using the previously constructed carbohydrate calibration curves.

### 2.5. Statistical analysis

All the results are expressed as mean ± SD. An ANOVA was performed for the regression with a 95% confidence limit in the Microsoft Office Excel program.

## 3. Results

### 3.1. Plates revealed

**Figure 2:**
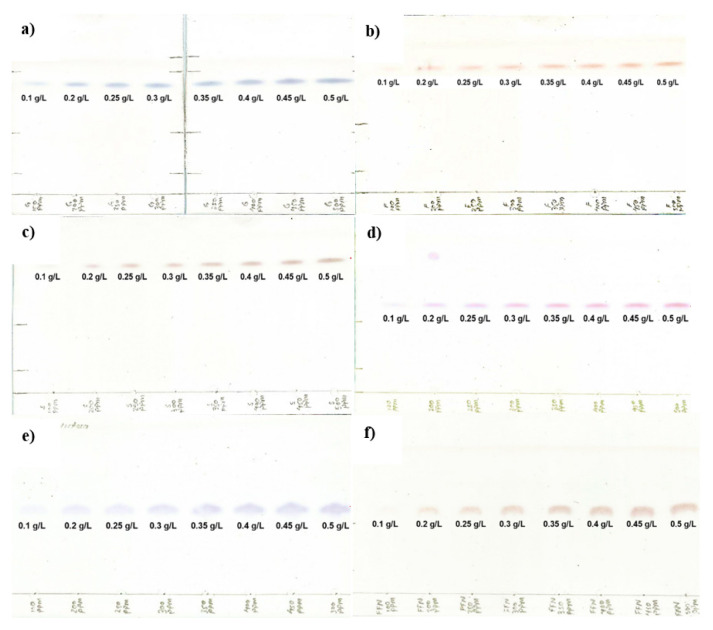
TLC plates with the calibration curves of the carbohydrate standards. a) glucose, b) fructose, c) sucrose, d) 1-kestose, e) nystose and, f) fructofuranosylnystose.

In the TLC assay of this study, it was obtained a coloration of the carbohydrates with a molecular weight similar to those obtained by Chand Bhalla et al. (2017) [19] (Figure 2).

Amber coloration was observed in the standard sucrose, blue coloration in glucose, and red coloration to the standards of fructose, 1-kestose, nystose, and fructofuranosylnystose.

The spots corresponding to the concentrations of 1 g/L of all the standards did not present a good sharpness or coloration, therefore, it was not possible to detect them with the image processing software and they were not included in the calibration curves.

### 3.2. Construction of calibration curves

**Figure 3:**
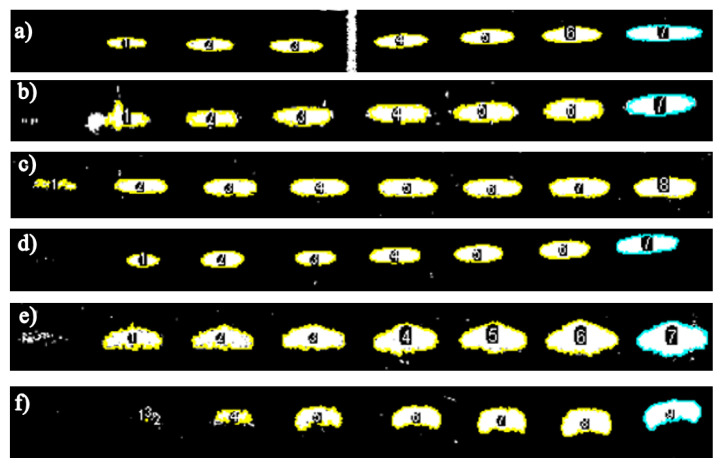
Segmentation of the calibration curves of the prebiotic standards with the ImageJ software. a) glucose, b) fructose, c) sucrose, d) 1-kestose, e) nystose and f) fructofuranosylnystose.

The areas of the spots of each compound were determined by digitizing and carrying out a segmentation of the resulting plates using the ImageJ software (Figure 3). The areas obtained from the spots of the different carbohydrates are shown in Table 1.

**Figure 4:**
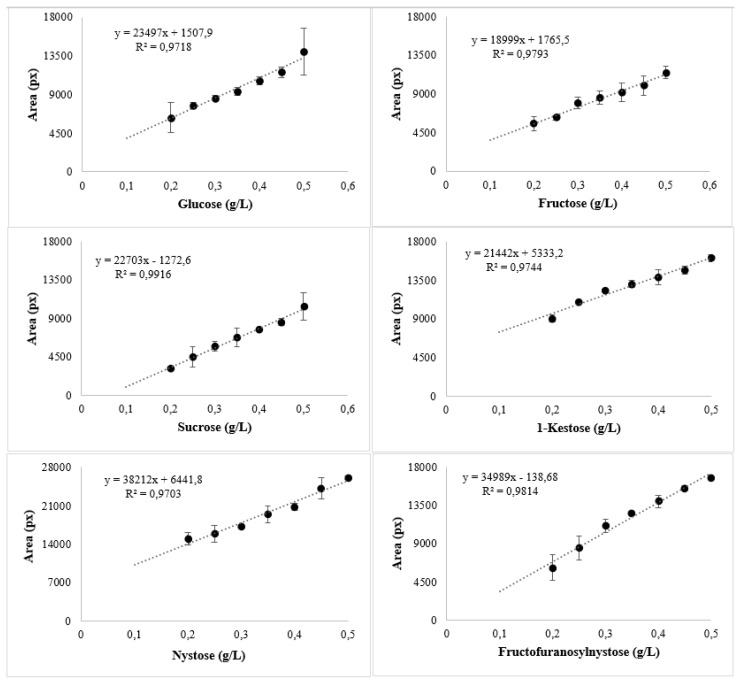
Calibration curves of carbohydrates standard tested.

The calibration curves were plotted using the average area of the pigmentations obtained in the different repeats, obtaining a coefficient of determination (R^2^) of 0.99 for sucrose, 0.97 for glucose, 0.97 for fructose, 0.97 for 1-kestose, 0.97 for nystose and 0.98 for fructofuranosylnystose. The formula of y = mx + b was established to determine the concentration of prebiotics in study samples, obtaining the following equations y = 22703x − 1272.6 for sucrose, y = 23497x + 1507.9 for glucose, y = 18999x + 1765.5 for fructose, y = 21442x + 5333.2 for 1-kestose, y = 38212x + 6441.8 for nystose and y = 34989x + 138.68 for fructofuranosylnystose (Figure 4). The ANOVA for the calibration models of the 6 carbohydrates studied showed that the sum of squares of the regressions is higher than that of the residuals in all models. In addition, the p-value less than 0.05 indicates that all models are significantly linear (Table 2).

### 3.3. Carbohydrate profile in food samples

Here, was possible to analyze samples of honey, garlic, and onion for the detection of simple carbohydrates and oligosaccharides using TLC chromatography (Table 3). Fructooligosaccharides (1-kestose and nystose) were found in the garlic (4 and 2.8 g/L) and onion (0.7 and 0.3 g/L), however, these prebiotics were not detected in honey samples.

## 4. Discussion

In the standardization of the TLC technique, glucose, sucrose, fructose, and fructooligosaccharides such as 1-kestose, nystose, and fructofuranosylnystose standards in concentrations of 0.1, 0.2, 0.25, 0.3, 0.35, 0.4, 0.45, and 0.5 g/L were analyzed, to establish the calibration curves for each standard and with these being able to graph the lines of the different carbohydrates using the ImageJ software comparing the concentration data in g/L and the area of the spots in pixels. ImageJ software is efficient for the semiquantitative determination of carbohydrates using Excel software (Microsoft) as a complement to the construction of the graphs of the calibration curves. With this data, it can be determined how effective the standardization was by placing a coefficient of determination (R^2^) in a range of 0 to 1, with 1 being the one with the highest precision. The software did not detect in the segmentation the low concentration standards due to the low saturation of the pigmentation colors. Therefore, the sensibility of this technique in the present work was in the range of 0.1–0.5 g/L.

All the coefficients of determination (R^2^) of the calibration curves constructed with the help of the ImageJ and Excel (Microsoft) software were greater than 0.95, therefore, these lines are acceptable to be used in determining the concentrations of carbohydrates present in samples of different substrates.

TLC coupled to image processing software has been used quantitatively for the determination of food colorants [15], main compounds of *Calendula officinalis* (faradiol, caffeic acid, gallic acid, and rutin) [20], and compounds of medicine tablets (acetaminophen, caffeine, and acetylsalicylic acid) [14]. In this study, a conventional office scanner was used to obtain chromatographic images and ImageJ software was used to analyze the images. Several authors have reported other alternatives for the obtention and processing of TLC chromatographic images, Machado & Fonseca (2019) [14] used a webcam (Logitech HD C270) to obtain chromatographic images and visual studio software for graphic processing. Saponar et al. (2008) [15] obtained the chromatographic images using a flatbed scanner (HP ScanJet 3970) and Macherey-Nagel TLC software for image analysis. Chromatographic images can be taken even with a smartphone camera with minimal specifications [21]. Thus, the application of TLC for quantitative determinations becomes a viable option when there is not enough capital available for the acquisition of specialized equipment or adequate infrastructure.

Thin-layer chromatography (TLC) is a simple, quick, reproducible, and economical method [22] for semiquantitative determination of carbohydrates mixtures in food samples in comparison with other analytical methods such as gas chromatography (GC), high-performance liquid chromatography (HPLC), anion-exchange liquid chromatography (AX-LC), enzymatic methods, electrochemical analysis, and flow injection analysis [23] because these methods commonly are expensive, the analysis is technically complicated and time-consuming [24]. Another alternative for carbohydrate determination is high-performance thin layer chromatography (HPTLC) which has great potential for qualitative and quantitative analysis by combining the simplicity of TLC with the automation and precision of densitometry [22, 23]. However, HPTLC still requires specialized equipment for quantitative determination. Thus, the conventional TLC method coupled with image processing software is a good option as an important tool for the quantification of carbohydrates or other compounds in food samples.

Zhang et al. (2009) [22] mentioned that is possible for the detection of various classes of carbohydrates such as monosaccharides, disaccharides, oligosaccharides, glycolipids, and other neutral and acid carbohydrates but it depends on the stationary phase and mobile phase used. Garlic and onion are plants used by ancient people as fructans-containing vegetables and also in Egypt were widely used in religious rites [24]. Garlic is known to have fructans that can be extracted by traditional techniques using solvents such as ethanol, methanol, and hot water. In a study conducted by Shalini et al. (2021) [27] methanol was found to be the best solvent for the extraction of fructans, which included kestose and nystose as well as inulin, which are fructans with a higher degree of polymerization. The presence of these types of FOS in garlic was qualitatively and quantitatively confirmed by the TLC method in the present study. Jovanovic-Malinovska et al. (2014) [28] evaluated various fruits and vegetables for the determination of prebiotic carbohydrates and they reported high concentrations of fructooligosaccharides in white onion while in garlic high levels of fructans were found (7.51 g/100 g fresh weight) using HPLC analysis. Pöhnl et al. (2017) [29] report glucose, fructose, sucrose, and diversity FOS content in onion (until DP 20) using high-performance anion exchange chromatography with pulsed amperometric detection (HPAEC-PAD), which has a sensitivity of 0.12 mg/L which allows the determination of the most carbohydrates present in onion. However, to obtain the benefits of the prebiotic carbohydrates it is necessary to higher concentrations, that are detected by the method purposed. This method has a sensitivity of 200 mg/L which avoids detecting carbohydrates below this concentration. Although the sample preparation for HPLC, HPAEC, or the other complex chromatography is more complicated, the sample preparation for TLC has a minimal requirement for filtration or centrifugation, moreover, the amount of sample required is small which allows the realization of various measurements in a lower time.

## 5. Conclusion

In the TLC technique, an efficient method was applied for all carbohydrates studied. The ImageJ software resulted in a useful software in the measurement of the areas of the spots in the TLC technique in the range of 0.2 to 0.5 g/L where the maximum software sensitivity is reached. The calibration curves were effective in the calculation of the carbohydrate concentration in all samples because the coefficient of determination was greater than 0.97 in all the curves. Interestingly, only garlic and onion presented prebiotics (FOS) and unfortunately, in this study, by the nature of the honey, there was no presence of prebiotic compounds.

In this study, it was possible to standardize the technique of TLC for the semiquantitative determination of carbohydrates (sucrose, glucose, fructose, 1-kestose, nystose, and fructofuranosylnystose), which could be useful, economical, and faster in research laboratories and the food industry as a preliminary analytic technique for the detection of these important compounds in food samples without the need to use specialized analytical equipment.

## Figures and Tables

**Table 1:** Areas obtained in the segmentation of the spots of carbohydrates standard by the ImageJ software.

Concentration g/L	Area (px)					
	Glu (RF: 0.83)	Fru (RF: 0.80)	Suc (RF: 0.77)	1-K (RF: 0.56)	N (RF: 0.52)	FN (RF: 0.47)
0.10	N/D	N/D	N/D	N/D	N/D	N/D
0.20	6313 ± 1750	5538 ± 792	3121 ± 106	9026 ± 415	15,041 ± 1161	6166 ± 1534
0.25	7701 ± 396	6214 ± 260	4510 ± 1216	10,942 ± 186	15,842 ± 1523	8493 ± 1361
0.30	8567 ± 287	7953 ± 682	5710 ± 552	12,293 ± 199	17,310 ± 164	11,096 ± 748
0.35	9335 ± 461	8553 ± 763	6790 ± 1110	13,073 ± 458	19,408 ± 1500	12,606 ± 232
0.40	10,600 ± 504	9179 ± 1070	7639 ± 123	13,814 ± 871	20,741 ± 655	14,014 ± 727
0.45	11,614 ± 611	9990 ± 1132	8590 ± 402	14,686 ± 458	24,228 ± 1908	15,580 ± 258
0.50	13,991± 2724	11,479 ± 753	10,353 ± 1609	16,030 ± 414	26,139 ± 371	16,796 ± 279

N/D: not Detectable; RF: retention factor (calculated as the ratio of the distance the spot moved above the origin to the distance the solvent front moved above the origin). Glu: glucose, Fru: fructose, Suc: sucrose, 1-K: 1-kestose, N: nystose, and FN: fructofuranosylnystose.

**Table 2:** ANOVA of calibration models for quantification of carbohydrates by TLC method.

Glucose					
	DF	SS	MS	F	P-value
Regression	1	700363525	700363525	2108.95111	7.14275E-09
Residuals	7	2324636.47	332090.925		
Total	8	702688161			
**Fructose**					
Regression	1	519328726	519328726	1667.4819	1.4422E-08
Residuals	7	2180114.26	311444.894		
Total	8	521508840			
**Sucrose**					
Regression	1	346954766	346954766	2091.51985	7.32237E-09
Residuals	7	1161205.03	165886.432		
Total	8	348115971			
**1-Kestose**					
Regression	1	1170813873	1170813873	516.44535	4.75401E-07
Residuals	7	15869437.3	2267062.48		
Total	8	1186683310			
**Nystose**					
Regression	1	2828890274	2828890274	790.529683	1.33946E-07
Residuals	7	25049321.1	3578474.45		
Total	8	2853939595			
**Fructofuranosylnystose**					
Regression	1	1111781491	1111781491	4757.65655	6.24723E-10
Residuals	7	1635778.11	233682.587		
Total	8	1113417269			

**Table 3:** Analysis by TLC for the carbohydrate determination in food samples.

Samples	Glucose (g/L)	Fructose (g/L)	Sucrose (g/L)	1-kestose (g/L)	Nystose (g/L)	Fructofuranosylnystose (g/L)
Honey	N/D	N/D	1043.3	N/D	N/D	N/D
Garlic	2.9	N/D	13.9	4	2.8	N/D
Onion	N/D	N/D	2.4	0.7	0.3	N/D

N/D = not detectable
